# Trajectories of Persistent Exposure to Self-Harm–Encouraging Websites and Subsequent Mental Health Outcomes in US Youth: Longitudinal Cohort Study

**DOI:** 10.2196/95200

**Published:** 2026-09-23

**Authors:** Kimberly J Mitchell, Ateret Gewirtz-Meydan, Victoria Banyard

**Affiliations:** 1 Crimes Against Children Research Center, University of New Hampshire, 10 West Edge Drive, Suite 106, Durham, NH, 03824, United States, 1 603-862-4533; 2 Faculty of Social Welfare and Health Sciences, School of Social Work, University of Haifa, Haifa, Israel; 3 School of Social Work, Rutgers University, New Brunswick, NJ, United States

**Keywords:** self-harm, suicidal ideation, self-injury, disordered eating, adolescents, young adults, online content, websites, longitudinal study

## Abstract

**Background:**

Self-harm, including suicidal thoughts, self-injurious behavior, and disordered eating, is a major public health concern among adolescents and young adults. Youth increasingly encounter self-harm–related content online, including websites that explicitly encourage harmful behaviors. Although exposure to such content has been linked to poorer mental health, most research is cross-sectional and has not examined longitudinal exposure trajectories. Whether persistent exposure represents a distinct digital risk pattern remains unclear.

**Objective:**

This study examined longitudinal trajectories and correlates of exposure to self-harm–encouraging websites over 1 year and tested whether persistent exposure was associated with subsequent suicidal ideation, self-injury, and disordered eating.

**Methods:**

We analyzed data from 2197 US youth aged 13 to 22 years who completed waves 2 and 4 of Project Lift Up, a national longitudinal cohort study. Website exposure was categorized into 4 trajectories: none, desist (wave 2 only), new (wave 4 only), and persistent (both waves). Categories reflected reported exposure at the 2 assessment waves rather than the frequency or continuity of website use between assessments. Logistic regression models estimated associations between exposure trajectory and wave 4 suicidal ideation, self-injury, and disordered eating, restricting analyses to participants without the respective outcome at wave 2. Models were adjusted for demographics and wave 2 depression or anxiety symptoms and other mental health indicators.

**Results:**

Overall, 26.5% (583/2197) reported exposure to self-harm–encouraging websites at wave 2, and 18.5% (407/2197) reported persistent exposure. Privacy concerns, embarrassment, curiosity, and lack of offline support were associated with greater odds of persistence (adjusted odds ratios [aORs] 1.84‐3.23), whereas accidental exposure was associated with lower odds of persistence (aOR 0.28, 95% CI 0.19‐0.41). Compared with no exposure, persistent exposure was associated with higher odds of suicidal ideation (aOR 1.88, 95% CI 1.24‐2.86; *P*=.003), self-injury (aOR 3.14, 95% CI 2.05‐4.80; *P*<.001), and disordered eating (aOR 2.46, 95% CI 1.54‐3.93; *P*<.001). Adjusted predicted probabilities showed a graded pattern, with persistent exposure associated with the highest predicted probabilities (suicidal ideation: 33.4% vs 21.3%; self-injury: 25.7% vs 10.2%; disordered eating: 11.7% vs 5.3%). New exposure was associated with increased odds of self-injury (aOR 2.32, 95% CI 1.30‐4.15; *P*=.004) but was not significantly associated with suicidal ideation or disordered eating. The desist trajectory was not significantly associated with outcomes.

**Conclusions:**

Persistent exposure to self-harm–encouraging websites may represent a distinct digital risk pattern associated with higher subsequent odds of suicidal ideation, self-injury, and disordered eating. Assessing patterns of repeated website exposure and youths’ motivations for seeking this content—not merely whether exposure occurred—may improve the identification of youth at heightened risk and inform digital prevention and clinical intervention strategies.

## Introduction

### Digital Exposure to Self-Harm–Encouraging Content

Self-harm among adolescents and young adults is a significant public health concern [1,2] and is associated with mental health disparities, impaired functioning, and elevated risk for injury and death [3,4]. Related self-harm behaviors, including disordered eating, also pose serious risks to psychological and physical well-being in this population [5-7]. In recent years, digital environments have become integral to the daily lives of youth and young adults and increasingly shape how they encounter information related to self-harm and extreme weight control behaviors [8-10]. While some websites offer social connection, supportive communities, or even strategies to reduce self-harm [11-15], others directly encourage harmful behaviors by normalizing self-injury, presenting suicide as a viable option, or promoting extreme dieting practices, including not eating or inducing vomiting to lose weight [14,16-18]. Exposure to these websites is associated with poorer mental health, lower levels of well-being, and lower likelihood of seeking professional help [19-22].

Youth arrive at self-harm websites that encourage suicidal behavior, self-injury, or disordered eating for varied reasons. Some intentionally seek them out to maintain privacy, avoid judgment, or explore self-harm–related or weight-related questions they feel unable to discuss with others [18]. Others encounter these sites unintentionally through misdirected searches or algorithmic recommendations. Although exposure to such content is well documented [10,20], it remains unclear whether most encounters are isolated or whether some youth continue returning across time. This distinction is important because persistent exposure may reflect ongoing vulnerability or difficulty accessing effective coping resources and may reinforce maladaptive emotional and behavioral patterns, including self-harm and disordered eating or may serve as a behavioral marker of youth experiencing persistent distress. Most existing research relies on cross-sectional assessments [10,18,19], limiting understanding of whether exposure persists, ceases, or emerges over time. Little is known about how prior motivations shape later website exposure, how demographic and mental health characteristics relate to persistence, or whether persistence is associated with subsequent behaviors such as thoughts of suicide, self-injurious behavior, and disordered eating.

### Current Study

The current study addresses these gaps by examining longitudinal patterns of exposure to self-harm–encouraging websites across a 1-year period. Website exposure was assessed at waves 2 and 4 but not at wave 1; therefore, wave 2 served as the analytic baseline for the longitudinal analyses, and exposure trajectories were defined across waves 2 and 4. Participants were classified as having no exposure at either wave (none), exposure at wave 2 only (desist), exposure at wave 4 only (new), or exposure at both waves (persistent). These trajectory groups reflect reported exposure at 2 assessment points rather than the frequency or continuity of website use between assessments. First, we assessed whether motivations for prior (wave 2) exposure were associated with persistent website exposure over time. Second, we examined demographic and wave 2 mental health characteristics associated with persistent exposure. Third, we tested whether distinct exposure trajectories (none, desist, new, persistent) were associated with suicidal ideation, self-injurious behavior, and disordered eating at follow-up, restricting analyses to participants without the respective outcome at wave 2. By integrating correlates and outcomes of persistent website exposure, this study provides longitudinal evidence on patterns of reported website exposure over time and how exposure reported at both waves is associated with subsequent mental health outcomes among adolescents and young adults.

## Methods

### Participants

*Project Lift Up* is a national longitudinal study of US youth and young adults designed to examine bystander behaviors and other experiences related to self-directed violence. Eligibility criteria included being 13 to 22 years of age at baseline, living in the United States, and proficiency in English at baseline. To recruit a diverse sample, demographic quotas were used based on age, gender identity, and sexual orientation. A total of 4981 participants, aged 13 to 22 years, were recruited between June 13, 2022, and October 30, 2023. Wave 2 was collected approximately 6 months after wave 1 between January 13, 2023, and February 26, 2024; wave 3 was collected between June 13, 2023, and November 19, 2024; and wave 4 was collected between December 15, 2023, and May 8, 2025. Although recruitment occurred on a rolling basis, participants completed wave 4 approximately 1 year after wave 2 according to the study protocol.

The present study used waves 2 and 4 because exposure to self-harm–encouraging websites was assessed at those waves but not at wave 1. Baseline demographic characteristics were drawn from wave 1, and wave 2 mental health measures served as the analytic baseline for the longitudinal models. The qualifying cohort included 3360 youth, of whom 2197 completed both wave 2 and wave 4 surveys and comprised the longitudinal cohort. Because Project Lift Up was designed to recruit a diverse cohort rather than a nationally representative sample, prevalence estimates should not be interpreted as representative of all US youth. Comparisons of participants retained in the analytic sample with those not retained from the qualifying cohort are presented in Table S1 in Multimedia Appendix 1.

### Procedures and Ethical Considerations

Participants were recruited through study advertisements on social media, including Facebook and Instagram, which linked eligible individuals to a secure, closed survey website. Those who were eligible were redirected to an assent (ages 13‐17 years) or consent (ages 18‐22 years) form to indicate their willingness to participate in the survey before continuing with the main survey. Youth who were not eligible were redirected to a web page that included links to general resources for youth. Participants were given a US $15 Amazon gift code for completing waves 1‐3 (with wave 3 participants receiving a US $5 early completer bonus for finishing the survey within the first 24 hours after being invited); participants who completed wave 4 received US $20.

We used demographic quotas to promote a diverse sample. Once the targeted number of youth in a particular group had been achieved (eg, aged 18‐22 years, sexual minority girls), any subsequent youth in this group who would have been eligible were classified as ineligible. Multiple fraud protection and detection procedures were used to ensure the quality of the responses, including age verification, reverse lookup of IP addresses and phone numbers, review of names and addresses, time to survey completion, and direct participant outreach, as needed.

The protocol was reviewed and approved by the University of New Hampshire Institutional Review Board. A waiver of caregiver permission was requested and granted by the Institutional Review Board for minors because requiring caregiver consent could potentially place youth in situations where their sexual experiences and/or sexual attraction could be unintentionally disclosed to their parents. Appropriate mechanisms were in place to protect youth participants, including access to a team clinician.

### Measures

#### Exposure to Self-Harm–Encouraging Websites

The primary exposure was self-reported exposure to websites that participants perceived as encouraging suicidal behavior, self-injury, or disordered eating or extreme weight-control behaviors. The primary analyses used a composite measure of exposure to any of these website types because they share the common characteristic of encouraging harmful self-directed behaviors; domain-specific analyses were conducted as supplementary analyses.

At waves 2 and 4, participants were asked whether they had intentionally gone to or seen a website that encouraged people to (1) kill themselves, (2) cut or hurt themselves, or (3) not eat or throw up so they could lose weight. Response options were yes or no. Participants were not provided with examples of specific websites or instructed whether social media platforms should or should not be considered. Accordingly, responses reflect participants’ own interpretation of websites containing content that encouraged these behaviors. Because website exposure was assessed only at waves 2 and 4, four exposure trajectories were defined: none (no exposure at either wave), desist (exposure at wave 2 only), new (exposure at wave 4 only), and persistent (exposure at both waves). These trajectory groups represent reported exposure at 2 assessment points rather than the frequency or continuity of website use between assessments.

#### Reported Motivations for Website Exposure

For each website type, participants who reported exposure were asked to indicate why they had visited that website. Response options included (1) some people do not know about my feelings (about losing weight, self-harm, or suicide, depending on the website type) and they might find out if I asked them my question; (2) privacy is important to me; I do not want anyone to know what I was searching for; (3) I was curious and wanted to learn more about the issue; (4) I was embarrassed to ask someone or admit I did not know; (5) I do not know anyone offline who could answer my specific questions; (6) someone I know suggested I go there; (7) I saw it by accident; and (8) I was worried about a friend or family member. Participants could select all that applied. Responses 1 to 6 were also combined to create a variable reflecting any reported intentional motivation for visiting a self-harm-encouraging website.

#### Mental Health

Depression and anxiety were measured at waves 2 and 4 with 8 items from the American Psychiatric Association’s DSM-5-TR Self-Rated Level 1 Cross-Cutting Symptom Measure - Child Age 11‐17 [23] assessing the frequency of symptoms, such as anxiety, sadness, physical discomfort, and sleep disturbances during the past 2 weeks.

Suicidal ideation was measured at waves 2 and 4 with 1 item from the DSM-5-TR Self-Rated Level 1 Cross-Cutting Symptom Measures for Ages 11‐17 [23]: “In the past two weeks, have you thought about killing yourself or dying by suicide?” Response options were yes, no, prefer not to answer.

Self-injury was measured at waves 2 and 4 using items drawn from the National Institute of Mental Health National Data Archive [24]. Participants were prompted “Thinking of things you may have done to yourself on purpose, have you done any of the following in the past two weeks …” (a) cut or carved your skin, (b) hit yourself, (c) pulled your hair, (d) burned your skin, and (e) done some other type of self-injury? Because the measure did not assess suicidal intent, these behaviors are referred to as self-injury throughout this paper.

Disordered eating was measured with items from the Centers for Disease Control and Prevention Youth Risk Behavior Surveillance System [25] questionnaire capturing fasting of 24 hours or longer, diet pills without doctor’s advice, and vomiting or taking laxatives to lose weight or keep from gaining weight, in the past 30 days.

Mental health outcomes were assessed using brief self-report items drawn from established assessment instruments and surveillance systems. These measures were selected to minimize participant burden in this large longitudinal cohort and were not intended to provide clinical diagnoses.

#### Demographic Characteristics

All demographic characteristics were measured at wave 1 and included race, ethnicity, sexual identity, gender identity, sex assigned at birth, age, and family income.

### Data Analysis

#### Overview

All analyses were conducted using StataNow/SE (version 19.5) [26]. The analytic sample included participants who completed both wave 2 and wave 4 surveys (longitudinal cohort; N=2197). Because website exposure was first assessed at wave 2, exposure trajectories were defined using wave 2 and wave 4 responses. Participants were classified into 4 mutually exclusive groups: none, indicating no exposure at either wave; desist, indicating exposure at wave 2 only; new, indicating exposure at wave 4 only; and persistent, indicating exposure at both waves. These trajectory categories reflect reported exposure at 2 assessment points and do not capture the frequency, duration, or continuity of website use between assessments. Wave 1 demographic characteristics were treated as baseline characteristics, whereas wave 2 mental health measures served as the analytic baseline for the longitudinal exposure and outcome models. Because the website exposure items used different reference periods at the 2 assessments (lifetime at wave 2 and past year at wave 4), the trajectory categories should be interpreted as patterns of reported exposure across the 2 assessment periods rather than definitive patterns of initiation, cessation, or continuous exposure.

Attrition analyses compared participants retained in the analytic cohort with those not retained from the qualifying cohort with respect to wave 1 demographic and mental health characteristics. Among participants completing wave 2, additional analyses compared those who did and did not complete wave 4 on wave 2 mental health characteristics and self-harm–encouraging website exposure. The results are presented in Table S1 in Multimedia Appendix 1.

The analyses reported in this paper were not preregistered.

#### Descriptive Analyses

We first described sociodemographic characteristics of the longitudinal cohort overall and by 4-category website exposure trajectory (none, desist [wave 2 only], new [wave 4 only], persistent [wave 2 and wave 4]). Categorical variables were summarized using frequencies and percentages. Group differences across trajectory categories were evaluated using *χ*^2^ tests. We then characterized exposure type and trajectory categories across waves. For each website type (any self-harm-encouraging website, suicide-encouraging, cutting/self-injury, and eating/weight-control), we calculated wave 2 and wave 4 prevalence and classified longitudinal exposure into four mutually exclusive categories (none, desist, new, persistent). Unadjusted odds ratios (ORs) for the association between wave 2 and wave 4 exposure were estimated using logistic regression.

#### Correlates of Persistent Website Exposure

To identify factors associated with persistent exposure (wave 2 and wave 4 vs all other trajectories), we estimated multivariable logistic regression models. Covariates included wave 1 demographic characteristics and wave 2 mental health characteristics—depression or anxiety symptoms, suicidal ideation, self-injury, and disordered eating. Wave 2 mental health characteristics were selected because they characterized participants at the beginning of the exposure trajectory period. Adjusted odds ratios (aORs) with 95% CIs are reported. To improve interpretability, we estimated adjusted predicted probabilities (average marginal predictions) from fitted models using postestimation margins. For binary predictors, predicted probabilities are shown for each category. For continuous predictors, predicted probabilities are presented at selected values (eg, age 14‐22; depression or anxiety score 0‐5).

#### Reported Motivations and Persistent Exposure

Among participants who reported any self-harm–encouraging website exposure at wave 2 (n=583), we examined whether reported motivations for wave 2 website exposure were associated with persistence at wave 4 using multivariate logistic regression. Models were adjusted for sex assigned at birth, gender minority identity, sexual minority identity, and depression or anxiety symptoms at wave 2.

#### Website Exposure Trajectories and Mental Health Outcomes

To examine whether exposure trajectory was associated with subsequent mental health outcomes, we estimated separate logistic regression models for (1) suicidal ideation, (2) self-injury, and (3) disordered eating at wave 4. Each model was restricted to participants without the outcome at wave 2. The primary exposure was 4-category website exposure trajectory (reference: none). Models were adjusted for baseline demographics (sex assigned at birth, gender minority identity, sexual minority identity, low income, age) and wave 2 mental health indicators (depression or anxiety and the other wave 2 mental health outcomes). Adjusted odds ratios with 95% CIs are presented. Adjusted predicted probabilities were estimated using postestimation margins.

#### Sensitivity and Domain-Specific Analyses

To examine specificity of associations, we conducted 3 domain-matched models (suicide-encouraging websites → suicidal ideation; cutting or self-injury websites → self-injury; eating or weight-control websites → disordered eating) and 6 cross-domain models examining each website exposure trajectory in relation to the 2 nonmatched mental health outcomes. These models followed the same wave 2 outcome-restricted approach and covariate adjustment strategy as the primary models.

All tests were 2-sided with statistical significance defined as *P*<.05. Analyses were conducted using a complete-case approach within each model. Participants were required to have nonmissing data on the exposure, outcome, and covariates included in that specific analysis. For longitudinal trajectory analyses, the analytic cohort was restricted to participants who completed both wave 2 and wave 4 surveys (N=2197). For outcome models, samples were further restricted to participants without the outcome at wave 2 and with complete data on model variables, resulting in outcome-specific analytic sample sizes. Missing survey responses coded as nonresponse (eg, “prefer not to answer” or item nonresponse) were treated as missing and excluded from regression models; participants who did not complete a wave were excluded from longitudinal analyses by design.

## Results

### Sample Characteristics

**Figure 1.**
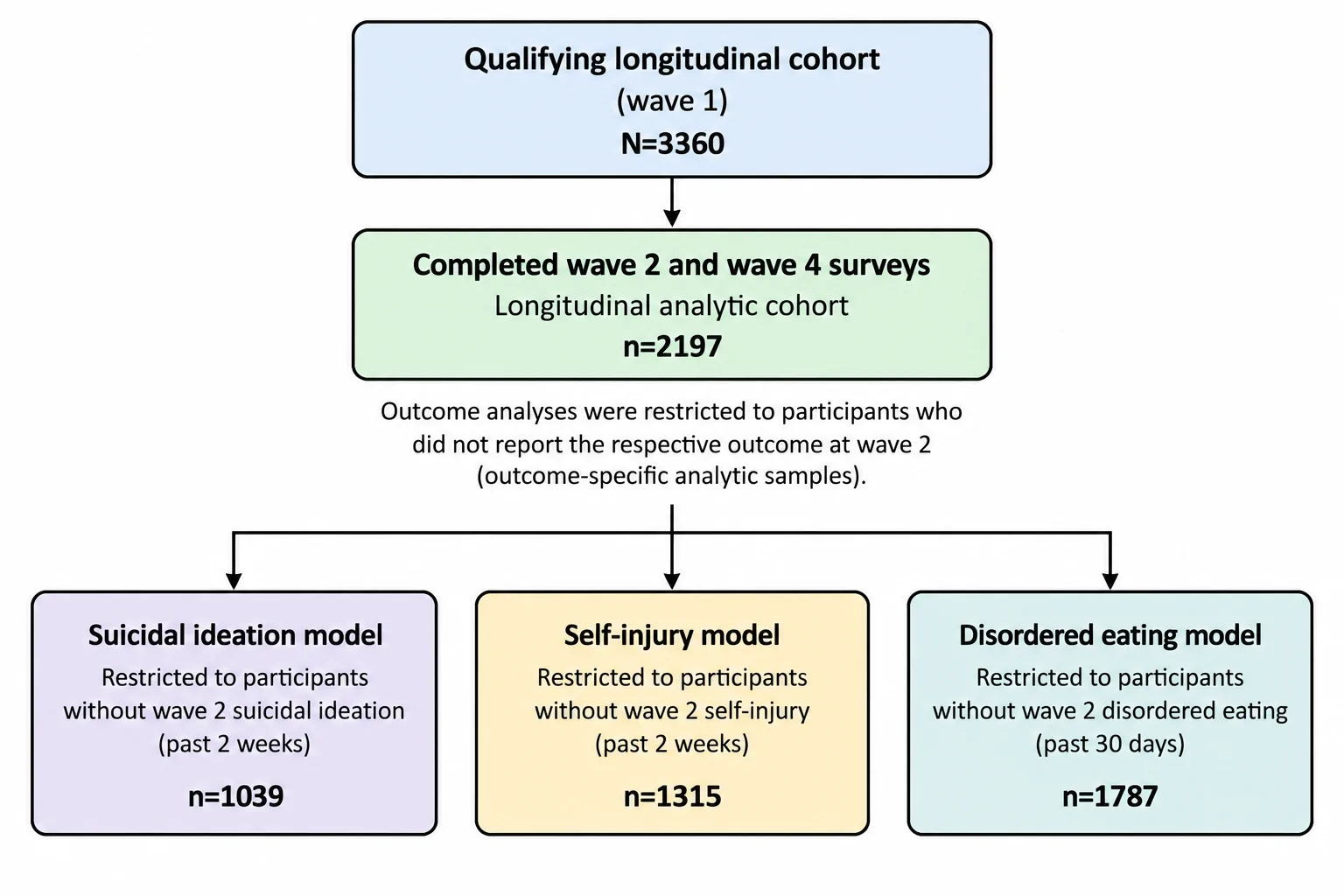
Participant flow diagram for the qualifying longitudinal cohort and outcome-specific analytic samples.

The qualifying cohort included 3360 youth, of whom 2197 completed both wave 2 and wave 4 surveys and comprised the longitudinal cohort (Figure 1). Overall, 26.5% (n=583) reported exposure to any self-harm–encouraging website at wave 2 (Table 1). Across both waves, 18.5% (n=407) demonstrated persistent exposure, 8.0% (n=176) reported exposure at wave 2 only (desist), and 7.7% (n=169) reported new exposure at wave 4. The remaining 65.8% (n=1445) reported no exposure at either wave.

**Table 1.** Self-harm–encouraging website exposure by website trajectory across waves 2 and 4 (N=2197).^a^

Website type	Any		Unadjusted odds of wave 4 exposure	None, n (%)	Persistent, n (%)	Desist, n (%)	New, n (%)
	Wave 2, n (%)	Wave 4, n (%)					
Any	583 (26.5)	576 (26.2)	19.77^b^	1445 (65.8)	407 (18.5)	176 (8.0)	169 (7.7)
Website that encouraged people to…							
Kill themselves	230 (10.6)	263 (12.0)	24.98^b^	1813 (84.2)	143 (6.6)	82 (3.8)	115 (5.3)
Cut or hurt themselves	237 (10.9)	281 (12.8)	29.32^b^	1813 (83.9)	155 (7.2)	76 (3.5)	117 (5.4)
Not eat or throw up to lose weight	427 (19.6)	425 (19.3)	21.32^b^	1604 (74.1)	279 (12.9)	142 (6.6)	139 (6.4)

a Trajectory percentages are based on participants with valid exposure responses at both waves; domain-specific Ns were 2153 for suicide-encouraging websites, 2161 for cutting or self-injury websites, and 2164 for eating or weight-control websites because of item nonresponse.

b All unadjusted odds ratios *P*<.001.

As shown in Table 2, persistent exposure differed significantly across several sociodemographic characteristics. Youth in the persistent group were more likely to be female (330/407, 81.1% vs 864/1445, 59.8% in the no exposure group; *P*<.001) and to identify as a gender minority (227/407, 55.8% vs 406/1,445, 28.1%; *P*<.001) or sexual minority (314/407, 77.1% vs 737/1445, 51.0%; *P*<.001). Age distribution did not significantly differ across trajectory groups (*P*=.08). Racial and ethnic differences were modest; White youth were somewhat overrepresented in the persistent group (325/407, 79.9%; *P*=.001). Family income did not significantly differ by trajectory (*P*=.22).

**Table 2.** Characteristics of youth by self-harm-encouraging website exposure trajectory (N=2197).

Characteristic	All participants (N=2197), n (%)	None (n=1445), n (%)	Desist (wave 2 only) (n=176), n (%)	New (wave 4 only) (n=169), n (%)	Persistence (waves 2 and 4) (n=407), n (%)	*χ*^2^ (*df*)	*P* value
Age (y)							
13‐17	1212 (55.2)	817 (56.5)	100 (56.8)	94 (55.6)	201 (49.4)	6.81 (3)	.08
18‐22	985 (44.8)	628 (43.5)	76 (43.2)	75 (44.4)	206 (50.6)	—^a^	—
Sex assigned at birth							
Male	704 (32.0)	543 (37.6)	57 (32.4)	50 (29.6)	54 (13.3)	92.38 (9)	<.001
Female	1421 (64.7)	864 (59.8)	114 (64.8)	113 (66.9)	330 (81.1)	—	—
Intersex	18 (0.8)	7 (0.5)	2 (1.1)	2 (1.2)	7 (1.7)	—	—
Prefer not to answer	54 (2.5)	31 (2.1)	3 (1.7)	4 (2.4)	16 (3.9)	—	—
Gender identity							
Cisgender boy or man	539 (24.5)	429 (29.7)	39 (22.2)	31 (18.3)	40 (9.8)	72.31 (3)	<.001
Cisgender girl or woman	870 (39.6)	604 (41.8)	61 (34.7)	58 (34.3)	147 (36.1)	8.75 (3)	.03
Nonbinary	392 (17.8)	198 (13.7)	34 (19.3)	40 (23.7)	120 (29.5)	58.70 (3)	<.001
Transgender girl or woman	89 (4.1)	58 (4.0)	10 (5.7)	12 (7.1)	9 (2.2)	8.80 (3)	.03
Transgender boy or man	250 (11.4)	103 (7.1)	26 (14.8)	23 (13.6)	98 (24.1)	93.83 (3)	<.001
Genderqueer	165 (7.5)	76 (5.3)	11 (6.3)	15 (8.9)	63 (15.5)	48.60 (3)	<.001
Genderfluid	153 (7.0)	83 (5.7)	19 (10.8)	16 (9.5)	35 (8.6)	10.62 (3)	.01
Agender	92 (4.2)	54 (3.7)	7 (4.0)	4 (2.4)	27 (6.6)	8.22 (3)	.04
Questioning	138 (6.3)	87 (6.0)	11 (6.3)	12 (7.1)	28 (6.9)	0.61 (3)	.89
* *Any gender minority identity	790 (36.0)	406 (28.1)	74 (42.1)	83 (49.1)	227 (55.8)	123.71 (3)	<.001
Sexual identity							
Gay	170 (7.7)	96 (6.6)	15 (8.5)	16 (9.5)	43 (10.6)	7.84 (3)	.05
Lesbian	141 (6.4)	69 (4.8)	10 (5.7)	20 (11.8)	42 (10.3)	25.22 (3)	<.001
Bisexual	507 (23.1)	298 (20.6)	51 (29.0)	44 (26.0)	114 (28.0)	14.77 (3)	.002
Heterosexual	881 (40.1)	696 (48.2)	58 (32.9)	41 (24.3)	86 (21.1)	121.51 (3)	<.001
Queer	179 (8.1)	85 (5.9)	21 (11.9)	14 (8.3)	59 (14.5)	35.20 (3)	<.001
Polysexual, omnisexual, sapiosexual, or pansexual	173 (7.9)	103 (7.1)	20 (11.4)	19 (11.2)	31 (7.6)	6.74 (3)	.08
Demisexual	73 (3.3)	36 (2.5)	9 (5.1)	6 (3.5)	22 (5.4)	10.39 (3)	.02
Asexual	177 (8.1)	109 (7.5)	14 (7.9)	14 (8.3)	40 (9.8)	2.25 (3)	.52
Questioning	92 (4.2)	70 (4.8)	2 (1.1)	1 (0.6)	19 (4.7)	11.32 (3)	.01
Any sexual minority identity	1298 (59.1)	737 (51.0)	122 (69.3)	125 (74.0)	314 (77.1)	117.08 (3)	<.001
Race^b^							
White	1613 (73.4)	1027 (71.1)	139 (79.0)	122 (72.2)	325 (79.9)	15.63 (3)	.001
Black or African American	270 (12.3)	189 (13.1)	22 (12.5)	21 (12.4)	38 (9.3)	4.14 (3)	.25
Asian	281 (12.8)	200 (13.8)	19 (10.8)	20 (11.8)	42 (10.3)	4.42 (3)	.22
More than one race	190 (8.7)	112 (7.7)	18 (10.2)	16 (9.5)	44 (10.8)	4.58 (3)	.21
Hispanic or Latino origin	455 (20.7)	296 (20.5)	36 (20.5)	45 (26.6)	78 (19.2)	4.25 (3)	.24
Family income							
Lower than the average family	600 (27.3)	367 (25.4)	58 (32.9)	47 (27.8)	128 (31.5)	11.82 (9)	.22
About the same as the average family	996 (45.3)	675 (46.7)	80 (45.5)	73 (43.2)	168 (41.3)	—	—
Higher than the average family	526 (23.9)	354 (24.5)	32 (18.2)	42 (24.9)	98 (24.1)	—	—
Prefer not to answer	75 (3.4)	49 (3.4)	6 (3.4)	7 (4.1)	13 (3.2)	—	—

a Not applicable.

b Multiple responses possible.

Attrition analyses are presented in Table S1 in Multimedia Appendix 1. First, among the 2686 participants who completed wave 2, we compared the 2197 participants who also completed wave 4 with the 489 participants who did not complete wave 4. Those who completed wave 4 were more likely to report wave 2 exposure to self-harm–encouraging websites than those who did not complete wave 4 (583/2197, 26.5% vs 101/489, 20.7%; *P*=.007), but the groups did not differ significantly in wave 2 depression or anxiety symptoms, suicidal ideation, self-injury, or disordered eating (all *P*>.13). Second, within the qualifying longitudinal cohort of 3360 participants, we compared the 2197 participants retained in the wave 2-wave 4 analytic sample with the 1163 participants who were not retained because they did not complete both waves. Retained participants were more likely to be female (*P*<.001), but the groups did not differ significantly by age, gender minority identity, sexual minority identity, family income, race, ethnicity, wave 1 depression or anxiety symptoms, suicidal ideation, or self-injury (all *P*>.05).

### Types of Self-Harm Website Exposure and Persistence

Exposure patterns varied by website type (Table 1). At wave 2, 230 (10.6%) participants reported visiting suicide-encouraging websites, 237 (10.9%) reported visiting cutting or self-injury websites, and 427 (19.6%) reported visiting eating or weight-control websites. Unadjusted odds of reporting exposure at wave 4 among participants exposed at wave 2 were elevated across all types (OR range 19.77‐29.32; all *P*<.001), indicating substantial persistence of reported exposure across the 2 assessment waves. Persistent exposure was observed among 143 participants (6.6%) for suicide-encouraging websites, 155 (7.2%) for cutting or self-injury websites, and 279 (12.9%) for eating or weight-control websites.

### Reasons for Wave 2 Exposure and Persistence

Among youth reporting wave 2 exposure (n=583), reporting 1 or more intentional motivations for visiting a self-harm-encouraging website was strongly associated with persistence at wave 4 (aOR 2.75, 95% CI 1.83‐4.14; *P*<.001; Table 3). Specific reported motivations, including privacy (aOR 3.23, 95% CI 1.98‐5.27; *P*<.001), concerns that others might find out about their feelings (aOR 2.07, 95% CI 1.39‐3.10; *P*<.001), curiosity (aOR 2.01, 95% CI 1.39‐2.92; *P*<.001), embarrassment (aOR 1.84, 95% CI 1.06‐3.18; *P*=.03), and not knowing whom to ask offline (aOR 2.47, 95% CI 1.54‐3.98; *P*<.001), were all independently associated with higher odds of persistence. In contrast, accidental exposure was associated with substantially lower odds of persistence (aOR 0.28, 95% CI 0.19‐0.41; *P*<.001).

**Table 3.** Associations between reported motivations for wave 2 website exposure and persistent exposure (n=583).

Reported motivation for wave 2 website exposure	Adjusted OR (95% CI)^a^	*P* value
Intentionally for self (any)	2.75 (1.83‐4.14)	<.001
Do not want people to know	2.07 (1.39‐3.10)	<.001
Privacy	3.23 (1.98‐5.27)	<.001
Curious	2.01 (1.39‐2.92)	<.001
Embarrassed	1.84 (1.06‐3.18)	.03
Do not know who to ask offline	2.47 (1.54‐3.98)	<.001
Someone suggested it	1.94 (0.77‐4.87)	.16
Saw by accident	0.28 (0.19‐0.41)	<.001
Worried about someone	1.55 (0.64‐3.74)	.33

a Models were adjusted for sex assigned at birth, gender minority identity, sexual minority identity, and wave 2 depression or anxiety symptoms.

### Correlates of Persistent Exposure

In multivariable models adjusting for demographic and wave 2 mental health variables (Table 4), female sex assigned at birth was associated with higher odds of persistent exposure (aOR 2.50, 95% CI 1.90‐3.30; *P*<.001). Gender minority identity (aOR 1.58, 95% CI 1.19‐2.10; *P*=.002) and sexual minority identity (aOR 1.61, 95% CI 1.18‐2.21; *P*=.003) were also independently associated with persistence. Older age was associated with increased odds of persistence (aOR per year 1.10, 95% CI 1.05‐1.16; *P*<.001). Higher wave 2 depression or anxiety symptoms were associated with greater likelihood of persistent exposure (aOR per unit 1.23, 95% CI 1.08‐1.40; *P*=.001). Wave 2 suicidal ideation (aOR 1.37, 95% CI 1.05‐1.79; *P*=.02), self-injury (aOR 1.38, 95% CI 1.05‐1.81; *P*=.02), and disordered eating (aOR 1.95, 95% CI 1.47‐2.57; *P*<.001) were also associated with persistence. Low income was not significantly associated with persistent exposure (*P*=.97). Adjusted predicted probabilities indicated a graded increase in persistent exposure from age 14 (14.2%) to age 22 (25.1%), and from low (score=0; 13.1%) to higher depression/anxiety symptoms (score=5; 28.1%).

**Table 4.** Correlates of persistent self-harm-encouraging website exposure across waves 2 and 4 among US youth and young adults (N=2197)^a^.

Construct	aOR^b^ (95% CI)	*P* value	Adjusted probability (%)^c^
Female sex assigned at birth	2.50 (1.90‐3.30)^d^	<.001	22.3 vs 10.9
Gender minority identity	1.58 (1.19‐2.10)^d^	.002	22.1 vs 15.7
Sexual minority identity	1.61 (1.18‐2.21)^d^	.003	20.6 vs 14.3
Low income	1.01 (0.78‐1.30)	.97	18.6 vs 18.5
Age (per 1-year increase)	1.10 (1.05‐1.16)^d^	<.001	14.2 (age 14) → 25.1 (age 22)^e^
Depression or anxiety symptoms (wave 2) (per 1-unit increase)	1.23 (1.08‐1.40)^d^	.001	13.1 (score 0) → 28.1 (score 5)^e^
Suicidal ideation (wave 2)	1.37 (1.05‐1.79)^d^	.02	20.3 vs 16.0
Self-injury (wave 2)	1.38 (1.05‐1.81)^d^	.02	20.2 vs 15.9
Disordered eating (wave 2)	1.95 (1.47‐2.57)^d^	<.001	26.6 vs 16.6

a Outcome: persistent exposure to self-harm–encouraging websites across waves 2 and 4 (yes vs no). Model: multivariable logistic regression (ORs shown); predicted margins from the fitted model. Model fit: LR *χ*²_9_=236.52; *P*<.001; pseudo *R*²=0.1123.

b aOR: adjusted odds ratio.

c Adjusted probabilities are predictive margins from the fitted model. For binary predictors, margins are shown for each category (yes vs no).

d *P*<.05.

e For continuous predictors, margins are shown at selected values to aid interpretation (age 14–22; depression/anxiety score 0–5).

### Website Exposure Trajectories and Mental Health Outcomes

#### Suicidal Ideation

Persistent exposure was associated with higher odds of suicidal ideation at wave 4 (aOR 1.88, 95% CI 1.24‐2.86; *P*=.003; Table 5). Adjusted predicted probability of ideation was 33.4% (95% CI 25.3‐41.4) among those with persistent exposure compared to 21.3% (95% CI 18.3‐24.2) among those with no exposure. Desist and new exposure patterns were not significantly associated with suicidal ideation.

**Table 5.** Associations between website exposure trajectory and wave 4 mental health outcomes^a^.

Outcome and exposure pattern	Adjusted OR (95% CI)	*P* value	Adjusted probability (%; 95% CI)
Suicidal ideation (n=1039)			
None	Ref	—^b^	21.3 (18.3‐24.2)
Desist	1.28 (0.70‐2.33)	.42	25.6 (15.0‐36.2)
New	1.48 (0.87‐2.52)	.14	28.5 (18.6‐38.3)
Persist	1.88 (1.24‐2.86)^c^	.003	33.4 (25.3‐41.4)
Self-injury (n=1315)			
None	Ref	—	10.2 (8.3‐12.1)
Desist	1.68 (0.90‐3.13)	.10	15.9 (8.2‐23.5)
New	2.32 (1.30‐4.15)^c^	.004	20.6 (12.0‐29.1)
Persist	3.14 (2.05‐4.80)^c^	<.001	25.7 (19.1‐32.4)
Disordered eating (n=1787)			
None	Ref	—	5.3 (4.0‐6.5)
Desist	1.77 (0.89‐3.49)	.10	8.8 (3.9‐13.7)
New	1.46 (0.72‐2.98)	.29	7.4 (3.0‐11.9)
Persist	2.46 (1.54‐3.93)^c^	<.001	11.7 (8.0‐15.5)

a Models were restricted to participants who did not report the respective outcome at wave 2 and adjusted for wave 1 demographic characteristics, wave 2 depression or anxiety symptoms, and the other wave 2 mental health indicators.

b Not applicable.

c *P*<.05.

#### Self-Injury

Both new (aOR 2.32, 95% CI 1.30‐4.15; *P*=.004) and persistent exposure (aOR 3.14, 95% CI 2.05‐4.80; *P*<.001) were associated with wave 4 self-injury among participants who did not report self-injury at wave 2. Predicted probability of self-injury increased from 10.2% (95% CI 8.3‐12.1) in the none group to 20.6% (95% CI 12.0‐29.1) in the new group and 25.7% (95% CI 19.1‐32.4) in the persistent group.

#### Disordered Eating

Persistent exposure was associated with disordered eating (aOR 2.46, 95% CI 1.54‐3.93; *P*<.001). Predicted probability was 11.7% (95% CI 8.0‐15.5) among persistently exposed youth compared with 5.3% (95% CI 4.0‐6.5) among those with no exposure. Associations for desist and new trajectories were not statistically significant. Across outcomes, adjusted predicted probabilities demonstrated a graded pattern, with persistent exposure consistently associated with the highest predicted probabilities.

### Domain-Specific and Cross-Domain Analyses

In matched-domain models (Table S2 in Multimedia Appendix 1), new and persistent exposure to cutting/self-injury websites was associated with higher odds of wave 4 self-injury, whereas the desist trajectory was not statistically significant. Desist, new, and persistent exposure to eating or weight-control websites were each associated with higher odds of wave 4 disordered eating. Suicide-encouraging website trajectories were not significantly associated with wave 4 suicidal ideation. In cross-domain models (Table S3 in Multimedia Appendix 1), suicide-encouraging website trajectories were associated with self-injury, with the strongest association observed for persistent exposure (aOR 5.12, 95% CI 2.83‐9.28; *P*<.001). Persistent suicide-encouraging website exposure was also associated with disordered eating. New cutting or self-injury website exposure was associated with suicidal ideation, and persistent cutting or self-injury website exposure was associated with disordered eating. Persistent eating or weight-control website exposure was associated with both suicidal ideation and self-injury. These supplementary findings suggest that associations were not limited to matched website-content and mental health domains and should be interpreted cautiously given the observational design and exploratory nature of these analyses.

## Discussion

### Principal Findings

This longitudinal study advances the understanding of how adolescents and young adults report exposure over time to websites that encourage self-harm behaviors, including suicidal thoughts and behaviors, self-injurious behavior, and extreme weight-control behaviors, and how distinct exposure trajectories were associated with subsequent mental health outcomes. Persistent exposure was associated with higher odds of all 3 wave 4 mental health outcomes, new exposure was associated with self-injury, and intentional motivations for wave 2 exposure were associated with exposure reported again at wave 4.

Exposure was not uniformly fleeting. Nearly 1 in 5 youth reported exposure at both waves, indicating that reported exposure to self-harm–encouraging websites was observed at both assessment points for a substantial proportion of youth. Importantly, persistent exposure was consistently associated with the highest odds of suicidal ideation, self-injury, and disordered eating 1 year later, even after adjusting for wave 2 mental health. Adjusted predicted probabilities demonstrated a graded pattern: youth with persistent exposure had substantially higher likelihood of subsequent ideation, self-injury, and disordered eating compared to those with no exposure. These findings suggest that repeated exposure reported at both waves may function as a behavioral marker of elevated clinical vulnerability.

New exposure was not uniformly benign. Youth who newly reported exposure to self-harm websites at wave 4 were also more likely to report self-injury, though not suicidal ideation or disordered eating. This differentiation underscores the value of distinguishing nonuse, desisting, new, and persistent patterns rather than collapsing exposure into a single binary measure. Different exposure trajectories may identify youth with different patterns of mental health risk.

A key finding concerns the associations of the reported motivations for wave 2 exposure. Youth who reported visiting these websites because of privacy concerns, embarrassment, curiosity, or lack of trusted offline support were substantially more likely to report exposure again at wave 4. These motivations reflect psychological factors well established in prior literature and associated with both self-harm and disordered eating behaviors [9]. That these reported motivations remained associated with persistent exposure after adjustment for baseline mental health suggests that understanding why youth seek out this content may provide additional information beyond exposure alone [8,17,18]. Conversely, accidental exposure was associated with lower odds of reporting exposure at wave 4. In addition, youth who visited because they were worried about someone else did not demonstrate elevated persistence, indicating a distinct, externally focused website exposure pathway.

Demographic and psychosocial characteristics further clarified vulnerability patterns. Youth assigned female at birth and those identifying as gender or sexual minorities were disproportionately represented among those with persistent exposure, even after multivariable adjustment. This is consistent with extensive literature documenting elevated rates of self-harm, disordered eating, internalizing symptoms, and minority stress–related distress among these populations [3,27]. Research also suggests that these groups of young people are more engaged in online spaces for identity exploration and support, which may increase exposure to both protective and harmful content [28]. Persistent exposure was also associated with higher wave 2 depression or anxiety symptoms and other self-harm–related behaviors, suggesting that persistent website exposure may serve as a marker of youth experiencing greater underlying psychological distress. Although baseline mental health was accounted for, the directionality of these associations cannot be determined, and residual confounding by unmeasured factors such as peer influences, family environment, or access to mental health care remains possible.

Importantly, domain-specific analyses indicated that some associations were content-specific. New and persistent exposure to cutting or self-injury websites was associated with subsequent self-injury, while desist, new, and persistent exposure to eating or weight-control websites were associated with subsequent disordered eating. Supplementary cross-domain analyses also identified associations between some website-content trajectories and outcomes outside the directly matched domain, including associations of suicide-encouraging website exposure with self-injury and of persistent eating or weight-control website exposure with both suicidal ideation and self-injury. Because these analyses were exploratory, they should be interpreted cautiously and warrant replication in future studies.

These findings contribute to a growing literature examining how digital environments intersect with mental health risk trajectories. Rather than conceptualizing online exposure as inherently harmful, this study suggests that persistence, intentionality, and user motivation are potential indicators of clinical relevance. Digital engagement patterns may therefore represent meaningful behavioral signals within broader suicide prevention and digital mental health frameworks. Although the present study focused on websites, the underlying concept of repeated exposure to self-harm–encouraging online content may also apply to contemporary digital environments, including social media platforms where such content may be encountered through search, sharing, or algorithmic recommendation. Future research should examine whether similar longitudinal exposure patterns emerge across these broader digital ecosystems and evaluate how platform-level interventions influence repeated exposure to harmful content.

### Limitations and Future Directions

This study is limited by its US-based sample, which may restrict generalizability to countries with different digital ecosystems or cultural norms surrounding help-seeking. Because Project Lift Up was designed to recruit a diverse cohort rather than a nationally representative sample, prevalence estimates should not be interpreted as representative of all US adolescents and young adults. All measures were self-reported, introducing possible social desirability bias and underreporting, especially given the potential secrecy surrounding self-harm behaviors and online activities. Website exposure was based on participants’ perceptions of websites that encouraged these behaviors; the survey did not provide examples or distinguish websites that actively promoted self-harm from those that discussed or provided information about these topics. In addition, participants were not instructed whether social media platforms should be included when responding, and exposure through social media, algorithmic feeds, or private messaging apps was not assessed separately.

The website exposure items also used different reference periods at the 2 assessments: wave 2 assessed lifetime exposure, whereas wave 4 assessed exposure during the past year. Consequently, the trajectory categories reflect patterns of reported exposure across the 2 assessment periods rather than definitive patterns of initiation, cessation, or continuous exposure. The trajectory categories also did not capture the frequency, duration, or intensity of website exposure between assessments. Some response options assessing motivations for website exposure, such as valuing privacy, may reflect broader motivations for seeking sensitive information online rather than motivations specific to visiting self-harm–encouraging websites. Although the mental health measures were drawn from established assessment sources, they consisted of brief self-report items and were not intended to provide clinical diagnoses. The psychometric performance of the self-injury items using the 2-week recall period in this study was not independently evaluated. Future research should map the broader digital ecology and examine how these environments interact with vulnerability, persistence, and clinical risk. Although models adjusted for wave 2 mental health indicators, causality cannot be inferred. Persistent exposure may reflect underlying vulnerability rather than independently causing subsequent symptoms. Residual confounding by unmeasured factors, including peer influences, family environment, access to mental health care, and co-occurring substance use, may also have contributed to the observed associations. Longitudinal designs incorporating finer-grained temporal measurement, ecological momentary assessment, or passive digital trace data may help clarify these mechanisms.

Although participants retained in the analytic cohort were more likely to be female than those not retained, they were otherwise similar with respect to demographic characteristics and wave 1 mental health measures, suggesting limited evidence of substantial attrition bias in the longitudinal analyses. Among wave 2 participants, retention through wave 4 was not associated with wave 2 mental health measures, although participants reporting wave 2 website exposure were more likely to complete wave 4. Differential attrition may therefore have affected prevalence estimates or associations involving website exposure.

### Clinical and Digital Health Implications

The findings highlight that persistent self-harm−encouraging website exposure may represent a meaningful behavioral marker of elevated clinical risk. For clinicians, digital behavior should be assessed not only in terms of whether youth have encountered harmful content but whether exposure has occurred repeatedly over time and the motivations for seeking such content. Youth who repeatedly report exposure to these websites, particularly for reasons related to secrecy, shame, or lack of offline support, may warrant more targeted assessment for suicidality, self-injury, and disordered eating.

These results support incorporating specific questions about persistent digital engagement into routine psychosocial assessments. For example, clinicians might ask whether youth have intentionally visited websites that encourage suicidal behavior, self-injury, or disordered eating; whether this exposure has been reported or experienced at more than 1 point in time; and what motivated them to seek this content. Although no widely adopted screening tools currently assess persistent exposure to self-harm–encouraging websites, these questions could be incorporated into existing psychosocial or suicide risk assessments. At a systems level, the findings suggest opportunities for digital health innovation, including supportive interventions that reduce repeated exposure to harmful online content across websites and other digital environments. Importantly, the Desist trajectory was not significantly associated with the examined outcomes, indicating that exposure reported at only wave 2 should not automatically be interpreted as evidence of elevated subsequent clinical risk.

### Conclusions

Persistent exposure to websites that encourage self-harm behaviors, including suicidal behaviors, self-injury, and disordered eating, may represent a distinct longitudinal digital risk pattern associated with elevated odds of subsequent mental health difficulties. New exposure was associated with increased odds of self-injury, whereas desisting patterns were not consistently associated with outcomes. Intentional, privacy-related, and other intentional motivations for wave 2 exposure were associated with persistence, highlighting the importance of understanding why youth seek out harmful digital content. Distinguishing between no exposure, wave 4-only exposure, wave 2-only exposure, and exposure reported at both waves provides a more precise framework for understanding digital risk. Identifying youth who repeatedly report exposure to these websites may help clinicians and digital health systems identify those at heightened risk while avoiding assumptions that website exposure itself caused subsequent mental health outcomes. A clearer understanding of these patterns can inform targeted prevention, early intervention, and digital mental health strategies.

## Supplementary material

10.2196/95200 Multimedia Appendix 1 Supplementary attrition and regression analyses.

## Abbreviations

aOR: adjusted odds ratio
OR: odds ratio
