# Supplementary material for: Trajectories of Persistent Exposure to Self-Harm–Encouraging Websites and Subsequent Mental Health Outcomes in US Youth: Longitudinal Cohort Study
Source: JMIR Ment Health. 2026 Sep 23;13:e95200. doi: 10.2196/95200 (PMC13600591; doi:10.2196/95200)
Supplement: Supplementary file 1 [file mental-v13-e95200-s001.docx]

**Multimedia Appendix 1: Supplementary Attrition and Regression Analyses**

**Table S1. Attrition Analyses for the Longitudinal Analytic Cohort**

| **Characteristic** | **Not retained (n=1163)** | **Retained (n=2197)** | **Test statistic** | **P value** |
| --- | --- | --- | --- | --- |
| **Wave 1 Characteristics of Participants Retained in the Wave 2-Wave 4 Analytic Cohort Versus Those Not Retained** | | | | |
| Age, years, mean (SD) | 17.50 (2.24) | 17.53 (2.23) | t=-0.30 (df=3358) | .77 |
| Age group: young adult, n (%) | 508 (43.7) | 985 (44.8) | χ²=0.41 (df=1) | .52 |
| Female sex assigned at birth, n (%) | 647 (55.6) | 1421 (64.7) | χ²=29.31 (df=3) | <.001 |
| Gender minority identity, n (%) | 416 (35.8) | 790 (36.0) | χ²=0.01 (df=1) | .91 |
| Sexual minority identity, n (%) | 649 (55.8) | 1298 (59.1) | χ²=3.35 (df=1) | .07 |
| Low-income household, n (%) | 309 (26.6) | 600 (27.3) | χ²=0.21 (df=1) | .65 |
| White, n (%) | 861 (74.0) | 1613 (73.4) | χ²=0.15 (df=1) | .70 |
| Black or African American, n (%) | 129 (11.1) | 270 (12.3) | χ²=1.04 (df=1) | .31 |
| Asian, n (%) | 139 (11.9) | 281 (12.8) | χ²=0.49 (df=1) | .49 |
| More than one race, n (%) | 120 (10.3) | 190 (8.7) | χ²=2.53 (df=1) | .11 |
| Hispanic/Latino ethnicity, n (%) | 264 (22.7) | 455 (20.7) | χ²=1.79 (df=1) | .18 |
| Racial or ethnic minority identity, n (%) | 520 (44.7) | 975 (44.4) | χ²=0.03 (df=1) | .85 |
| Depression/anxiety score, mean (SD) | 1.27 (0.69) | 1.25 (0.67) | t=0.64 (df=3358) | .52 |
| Lifetime suicidal ideation, n (%) | 921 (79.2) | 1723 (78.4) | χ²=0.27 (df=1) | .61 |
| Past-2-week suicidal ideation, n (%) | 413 (35.5) | 783 (35.6) | χ²=0.01 (df=1) | .94 |
| Lifetime self-injury, n (%) | 902 (77.6) | 1695 (77.1) | χ²=0.07 (df=1) | .79 |
| Past-year self-injury, n (%) | 738 (63.5) | 1404 (63.9) | χ²=0.07 (df=1) | .80 |
| **Wave 2 Characteristics of Participants Who Did and Did Not Complete the Wave 4 Survey** | | | | |
|  | Did not complete Wave 4 (n = 489) | Completed Wave 4 (n = 2,197) |  |  |
| Depression/anxiety score, mean (SD) | 1.90 (0.99) | 1.88 (1.00) | t=0.39 (df=2684) | .69 |
| Suicidal ideation, n (%) | 272 (55.6) | 1140 (51.9) | χ²=2.24 (df=1) | .13 |
| Self-injury, n (%) | 206 (42.1) | 859 (39.1) | χ²=1.53 (df=1) | .22 |
| Disordered eating, n (%) | 306 (62.6) | 1360 (61.9) | χ²=0.08 (df=1) | .78 |
| Any self-harm-encouraging website exposure, n (%) | 101 (20.7) | 583 (26.5) | χ²=7.29 (df=1) | .01 |

**Notes.** Values are *n* (%) unless otherwise indicated. *P* values are two-sided. The first section compares the 3360 participants in the qualifying longitudinal cohort according to inclusion in the Wave 2–Wave 4 analytic cohort. The second section is restricted to the 2686 participants who completed Wave 2 and compares those who did and did not complete Wave 4. The overall chi-square test for sex assigned at birth included the male, female, intersex, and prefer not to answer categories. Race categories were not mutually exclusive. SD = standard deviation.

**Table S2. Matched-Domain Website Exposure Trajectories Predicting Wave 4 Mental Health Outcomes**

| **Matched-domain model** | **N** | **Exposure trajectory** | **aOR (95% CI)** | **P value** | **Adjusted probability, % (95% CI)** |
| --- | --- | --- | --- | --- | --- |
| **Suicide-encouraging websites → suicidal ideation** | 1025 | None | Reference | — | 22.4 (19.7–25.1) |
|  |  | Desist | 2.12 (0.86–5.21) | .10 | 37.3 (17.3–57.3) |
|  |  | New | 1.85 (0.95–3.61) | .07 | 34.4 (20.3–48.4) |
|  |  | Persistent | 1.36 (0.68–2.70) | .39 | 28.0 (14.9–41.0) |
| **Cutting/self-injury websites → self-injury** | 1303 | None | Reference | — | 11.5 (9.7–13.3) |
|  |  | Desist | 2.25 (0.90–5.59) | .08 | 22.3 (7.3–37.3) |
|  |  | New | 3.88 (2.13–7.06) | <.001 | 32.8 (20.7–44.9) |
|  |  | Persistent | 2.94 (1.57–5.52) | .001 | 27.2 (15.7–38.7) |
| **Eating/weight-control websites → disordered eating** | 1772 | None | Reference | — | 5.3 (4.1–6.4) |
|  |  | Desist | 2.14 (1.05–4.37) | .04 | 10.5 (4.4–16.5) |
|  |  | New | 2.56 (1.34–4.91) | .005 | 12.2 (6.0–18.3) |
|  |  | Persistent | 2.53 (1.48–4.30) | .001 | 12.0 (7.3–16.8) |

**Note.** Each model was restricted to participants who did not report the respective outcome at Wave 2. Reference group for website exposure trajectory was no exposure at Wave 2 or Wave 4 (“None”). Models adjusted for sex assigned at birth, gender minority identity, sexual minority identity, low income, age, Wave 2 depression/anxiety symptoms, and the other Wave 2 mental health indicators. Adjusted probabilities were estimated using postestimation margins and represent average predicted probabilities holding covariates at their observed values. aOR indicates adjusted odds ratio; CI, confidence interval.

**Table S3. Cross-Domain Website Exposure Trajectories Predicting Wave 4 Mental Health Outcomes**

| **Website exposure → outcome** | **N** | **Trajectory** | **aOR (95% CI)** | **P value** | **Adjusted probability, % (95% CI)** |
| --- | --- | --- | --- | --- | --- |
| **Suicide-encouraging →**  **self-injury** | 1296 | None | Reference | — | 11.2 (9.4–13.0) |
|  |  | Desist | 3.03 (1.43–6.44) | .004 | 27.1 (13.1–41.0) |
|  |  | New | 3.45 (1.73–6.88) | <.001 | 29.6 (16.2–43.0) |
|  |  | Persistent | 5.12 (2.83–9.28) | <.001 | 38.1 (25.3–50.9) |
| **Suicide-encouraging → disordered eating** | 1762 | None | Reference | — | 5.9 (4.7–7.1) |
|  |  | Desist | 1.93 (0.79–4.70) | .15 | 10.6 (2.7–18.5) |
|  |  | New | 2.06 (0.99–4.29) | .05 | 11.2 (4.5–17.9) |
|  |  | Persistent | 1.99 (1.04–3.81) | .04 | 10.9 (5.2–16.6) |
| **Cutting/self-injury →**  **suicidal ideation** | 1030 | None | Reference | — | 22.0 (19.4–24.7) |
|  |  | Desist | 1.30 (0.48–3.57) | .61 | 26.8 (7.9–45.6) |
|  |  | New | 2.10 (1.16–3.82) | .02 | 36.7 (23.9–49.6) |
|  |  | Persistent | 1.87 (0.94–3.70) | .07 | 34.1 (19.7–48.5) |
| **Cutting/self-injury → disordered eating** | 1768 | None | Reference | — | 5.9 (4.7–7.1) |
|  |  | Desist | 2.23 (0.89–5.62) | .09 | 12.0 (2.9–21.1) |
|  |  | New | 1.22 (0.51–2.94) | .65 | 7.1 (1.6–12.5) |
|  |  | Persistent | 2.46 (1.31–4.63) | .01 | 13.0 (6.6–19.4) |
| **Eating/weight-control → suicidal ideation** | 1027 | None | Reference | — | 22.5 (19.6-25.3) |
|  |  | Desist | 1.07 (0.56–2.04) | .84 | 23.7 (12.8-34.5) |
|  |  | New | 0.81 (0.37–1.75) | .59 | 19.1 (7.8-30.4) |
|  |  | Persistent | 1.71 (1.07–2.74) | .03 | 32.8 (23.5-42.1) |
| **Eating/weight-control → self-injury** | 1305 | None | Reference | — | 11.7 (9.7–13.7) |
|  |  | Desist | 1.28 (0.64-2.54) | .48 | 14.4 (6.5–22.3) |
|  |  | New | 1.76 (0.91-3.41) | .09 | 18.7 (9.4–28.0) |
|  |  | Persistent | 2.44 (1.51-3.97) | <.001 | 24.0 (16.4–31.7) |

**Note.** Each model was restricted to participants who did not report the respective outcome at Wave 2. The reference category for website exposure trajectory was no exposure at Wave 2 or Wave 4 (“None”). Models adjusted for sex assigned at birth, gender minority identity, sexual minority identity, low income, age, Wave 2 depression/anxiety symptoms, and the other Wave 2 mental health indicators. Adjusted probabilities were estimated using postestimation margins and represent average predicted probabilities holding covariates at their observed values. aOR indicates adjusted odds ratio; CI, confidence interval.
